# Retrospective Analysis of Pseudotumor of the Hip: Clinical Management and Outcomes

**DOI:** 10.1111/os.70349

**Published:** 2026-07-09

**Authors:** Roberta Laranga, Luca Cevolani, Eric Lodewijk Staals, Anna Mammone, Laura Campanacci, Barbara Dozza, Davide Maria Donati, Giuseppe Bianchi

**Affiliations:** ^1^ Unit of 3rd Orthopedic and Traumatologic Clinic Prevalently Oncologic IRCCS Istituto Ortopedico Rizzoli, via Pupilli 1 Bologna Italy; ^2^ Department of Biomedical and Neuromotor Sciences (DIBINEM) Alma Mater Studiorum University of Bologna Bologna Italy

**Keywords:** hip arthroplasty revision, pseudotumor, revision outcome

## Abstract

**Background:**

Management of pseudotumors following hip arthroplasty remains a subject of debate. This study aims to assess implant survival in relation to clinical variables and compares the outcomes of one‐stage and two‐stage revision surgeries.

**Methods:**

We retrospectively analyzed 55 cases of pseudotumor surgically treated between 2004 and 2023 at a single tertiary care center. Patients underwent either one‐stage (44%) or two‐stage (29%) revision procedures. The following clinical and demographical information were collected: gender, age at diagnosis, body mass index (BMI) (calculated by dividing the patients' weight by their height squared solid mass), the American Society of Anaesthesiologist values (ASA), a diagnosis of pseudotumor identified by radiology and histological examination, time of diagnosis, surgery treatment, and follow‐up. Implant and patient survival were estimated using the Kaplan–Meier method.

**Results:**

The mean follow‐up was up to 67 months (95% CI 43.43–95.29), and the mean age at diagnosis was 56 years (95% CI 53–59). Revision strategy correlated with CRP levels (*p* = 0.013) and tumor size (*p* = 0.003). Ten complications (18%) occurred at a mean of 10 months, including wound issues (1), dislocations (4), abductor dysfunctions (4), and deep infection (1). Implant failure occurred in 3 cases (5%). Implant survival was 92% at 5 years (95% CI 0.68–0.97) and 80% at 10 years (95% CI 0.44–0.94). Fourteen patients died by final follow‐up; 2 deaths (14%) were surgery related. Overall patient survival was 83% at 5 years (95% CI 0.68–0.92) and 67% at 10 years (95% CI 0.48–0.80). Postoperative Harris Hip Score (*n* = 34) averaged 73.2 (95% CI 68.6–77.8).

**Conclusions:**

Surgery is recommended for pseudotumor, although the optimal approach remains argued. Our data support a two‐stage revision for large pseudotumors with elevated inflammatory markers. Revision surgery provides good function and low implant failure risk, but patient mortality remains significant due to fragility.

## Graphical abstract

The surgical revision treatment of patients affected by pseudotumor guarantees a good functional result with a low risk of failure of the new implant. However, pseudotumor requires a systematic and efficient surgical approach to be quickly and successfully resolved. We recommend a two‐stage approach in the presence of bigger lesions and ongoing infection to reduce the surgical time for each surgery and allow a more fitting design of a revision implant. Moreover, the increasing use of custom 3D‐printed implants supports the choice of a two‐stage procedure.

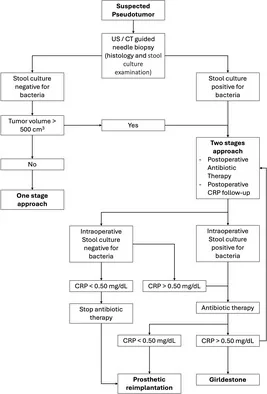

## 1 Introduction

Pseudotumor is a significant complication following total hip arthroplasty (THA), primarily associated with wear debris from implant bearing surfaces. Although the exact etiology remains unclear, it is generally understood to result from adverse tissue reaction involving lymphocyte infiltration and soft tissue necrosis due to debris releases in the periprosthetic environment [1, 2].

The reported prevalence of pseudotumor following THA varies significantly depending on implant type and patient population. In metal‐on‐metal (MoM) prostheses, the incidence ranges widely from 1% to 39%, with higher rates observed in younger, more active patients [3, 4, 5]. For metal‐on‐polyethylene (MoP) implants, the occurrence is lower, estimated between 0.5% and 5%, often linked to advanced wear and local inflammatory responses [4, 6]. Cases related to ceramic‐on‐ceramic (CoC) or ceramic‐on‐polyethylene (CoP) bearings are exceedingly rare, with incidences reported below 0.1%, although isolated case reports do exist [7].

Clinically, pseudotumor may be asymptomatic in up to 60% of cases [8, 9], but can progress to cause swelling, pain, soft tissue necrosis, and massive osteolysis, often necessitating revision surgery. Imaging modalities such as MRI and CT scans are critical for diagnosis, as pseudotumors can mimic soft tissue tumors or infections, making differential diagnosis and biopsy important steps [2].

The time to pseudotumor development after implantation typically ranges from 2 to 10 years, correlating with the amount and nature of wear debris released into the periprosthetic environment [3, 4]. Longitudinal studies have also reported that 10%–15% of patients undergoing revision surgery for pseudotumor experience significant postoperative complications, underscoring the need for optimized treatment strategies [9, 10].

Over the years, several bearing surfaces have been developed to minimize wear‐related complications, including metal‐on‐polyethylene (MoP), metal‐on‐metal (MoM), ceramic‐on‐ceramic (CoC), and ceramic‐on‐polyethylene (CoP) [11]. While pseudotumors are most frequently reported with MoM and MoP implants, rare cases have also been documented with CoC bearings, though incidence is comparatively lower [7].

Currently, there is no consensus on the optimal management of pseudotumors. The standard treatment consists of pseudotumor excision and revision of the prosthetic implant, which can be carried out in either one or two stages. However, single‐stage revision has been associated with a higher risk of postoperative complications [9, 12]. The two‐stage approach, although less commonly studied, may offer benefits in selected patients, but literature on its efficacy remains limited [12, 13]. Given the complexity of pseudotumor management, a multidisciplinary approach is often essential. Understanding the prognostic factors and treatment outcomes is crucial to improving clinical practice. Our study aims to identify key predictive factors influencing implant survival and to compare the clinical outcomes of one‐stage versus two‐stage revision procedures, contributing to establishing clearer treatment guidelines.

## 2 Material and Methods

### 2.1 Patients and Methods

This is a retrospective cohort study of 54 55 patients treated surgically from 2004 to 2023 for pseudotumor in a single tertiary centre (Istituto Ortopedico Rizzoli). Our institutional review board approved this retrospective study (CE‐AVEC 482/2022/Oss/IOR).

The main inclusion criteria were confirmed diagnosis of pseudotumor obtained before surgery, either by biopsy or by imaging. The presence of comorbidities preventing surgical treatment was the exclusion criteria of this study. Patients were included according to the following criteria: (i) histologically or radiologically confirmed diagnosis of pseudotumor prior to surgery; (ii) treatment with revision hip arthroplasty using either a one‐stage or two‐stage approach; and (iii) availability of clinical and follow‐up data. All included patients underwent either a one‐stage or two‐stage revision procedure. Exclusion criteria were: (i) presence of comorbidities precluding surgical treatment.

The following clinical and demographical information were collected: gender, age at diagnosis, body mass index (BMI) (calculated by dividing the patients' weight by their height squared solid mass), the American Society of Anaesthesiologist values (ASA), a diagnosis of pseudotumor identified by radiology and histological examination, time of diagnosis, surgical treatment, and follow‐up.

All patients were pre‐operative examined physically, with radiological imaging (CT scan or MRI), blood test (C‐Reactive Protein test value), and the type of bearing. Presence of infection was detected by pathological microbiology analysis. Histologic evaluation with biopsy was required for differential diagnosis with malignant tumors in bone and soft tissue.

Surgical treatment was selected according to the patient's general condition, infection status, implant stability, bone stock, soft‐tissue involvement, extent of pseudotumour or metallosis, feasibility of reconstruction, and expected functional benefit. One‐stage revision was preferred when infection was absent or considered adequately excluded, soft tissues were suitable, and stable definitive reconstruction was achievable. Two‐stage revision was selected when infection was suspected or confirmed, or when local conditions required staged debridement and temporary spacer implantation before definitive reconstruction. The Girdlestone procedure was reserved for selected patients in whom definitive reconstruction was not considered feasible or safe because of poor local or systemic conditions. Simil‐DAIR surgery was performed in cases suitable for pseudotumour excision and surface bearing component replacement, with retention of stable components. Hindquarter amputation was considered only as a salvage procedure in cases with extensive local involvement and no reasonable reconstructive option. Surgical treatment was classified as follows: one‐stage revision, two‐stage revision, Girdelstone procedure, Simil‐dair, and hind quarter amputation. Simil‐dair surgery was carried out as pseudotumor excision and surface bearing component replacement. All procedures were included in the descriptive analysis; however, only one‐stage and two‐stage revisions were included in comparative and survival analyses.

According to our institutional protocol, during the first surgical procedure (one‐stage revision or stage‐one of a two‐stage plan) the pseudotumour was not removed en bloc as an oncologic lesion. Instead, we carried out a selective debulking, consisting of decompression of cystic components and removal of necrotic pseudocapsule, while leaving in situ portions densely adherent to the abductor mechanism, capsule, or neurovascular structures. The extent of debridement was individualized intra‐operatively to avoid compromising hip stability and to reduce peri‐ and post‐operative haemorrhagic risk; additional tissue trimming at reimplantation (stage‐two) was performed only if safe and beneficial.

Following the first‐stage surgery, patients were placed on a restricted weight‐bearing protocol due to the use of temporary cement spacers and, in several cases, significant acetabular bone defects. Rehabilitation was individualized to maintain joint range of motion and muscle strength while minimizing load on the operated limb to prevent spacer dislocation or mechanical failure. Patients with stable spacers and minimal bone loss were allowed partial weight‐bearing, whereas those with extensive defects remained non‐weight‐bearing.

The median interval between the first and second‐stage surgeries was approximately 5 months (range 1–13 months). The timing of the second‐stage revision was based on normalization of C‐reactive protein (CRP) levels, absence of clinical or radiological signs of persistent inflammation or spacer‐related complications, and satisfactory soft tissue recovery. The second‐stage procedure consisted of spacer removal, extensive debridement, collection of intraoperative tissue samples for microbiological assessment, thorough irrigation, and implantation of the definitive reconstruction. Patients did not undergo second‐stage surgery if infection control was not achieved, if local soft‐tissue conditions remained inadequate, or if their general condition contraindicated further major reconstructive surgery. This approach aimed to optimize local tissue conditions and implant survival while balancing the risks associated with prolonged immobilization.

The postoperative protocol was the same for all patients. They were followed between 1, 3, 6 months and 1 year after revision, consisting of serial chest CTs and radiographs of the site of the tumors. Harris Hip score (HHS) was recorded after surgical treatment. A result over 70 points corresponded to a good state for the hip articulation [14].

### 2.2 Statistical Analysis

Descriptive statistics were presented as frequencies and percentages. Measures of association were estimated by the Chi‐square test or Fisher's exact test for association (contingency) between categorical variables. Patients' survival was defined as the time from the implant revision to death or the last follow‐up (study deadline, set for March 01, 2023). Implant survival was defined as the time from the implant revision to the time of implant failure due to post‐surgical complications or to the last follow‐up. We used the Kaplan–Meier method to estimate the survival over time. Follow‐up duration varied due to the long inclusion period (2004–2023). Differences in survival according to clinical variables were evaluated using the Log‐rank test. We performed the statistical analyses using STATA/SE version 18.0 Software (StataCorp LLC, College Station, TX, USA, 2023).

## 3 Results

### 3.1 Patient's Characteristics

We enrolled in this study a total of 55 cases of pseudotumor between 2004 and 2023. The mean follow‐up was 67 months (95% CI 43.43–95.29). Females represent 62% (34/55) of all enrolled patients, while men corresponded to 38% of all cases (21/55). The mean age at the surgery was 56 years (95% CI: 53–59), with a mean BMI of 26.45 kg/m^2^ (95% CI: 25–27.8). Twenty patients (36%) were classified as normal (BMI 18.5–21.9), 23 (42%) as overweight (BMI 25.0–29.9), 5 (9%) as Obesity 1st class (BMI 30.0–34.9), 5 (9%) as Obesity 2nd Class (BMI 35.0–39.9), and the last 2 patients (4%) as underweight (BMI < 18.5). C‐Reactive Protein (CRP) test value was collected to evaluate inflammatory status: 33/55 (67%) patients had an altered CRP value (cut‐off > 0.50 mg/dL), and 16/55 (33%) had in‐range value. We reported the American Society of Anaesthesiologist values (ASA), that corresponded to I for 3 patients (5%), II for 29 patients (53%), III for 21 patients (38%), and IV for the last 2 patients (4%). The evaluation of tumor size was performed by segmentation technique on MRI and was evaluated in 48 out of 55 cases. The mean tumor size was 573 cm^3^ (95% CI: 403–743). The demographic baseline of the included patients and the corresponding pre‐operative values are shown in Table 1.

**TABLE 1:** Demographic baseline and pre‐operative values of the included patients.

Variable	Category	*N* (%)
Gender	Male	21 (38)
	Female	34 (62)
Age	≤ 56	20 (36)
	> 56	35 (64)
Mean age 56 years (95% CI 53–59)		
BMI	Normal	19 (35)
	Overweight	24 (44)
	Obesity 1st class	5 (9)
	Obesity 2nd class	5 (9)
	Underweight	2 (4)
CRP	In range	16 (33)
	Altered	33 (67)
ASA	I	3 (5)
	II	29 (53)
	III	21 (38)
	IV	2 (4)
Mean tumor size (cm^3^) 573 (95% CI: 403–743)		

*Note:* 95% Interval of confidence (CI); body max index (BMI); CRP (C‐reactive Protein); American Society of Anaesthesiologist values (ASA).

**FIGURE 1:**
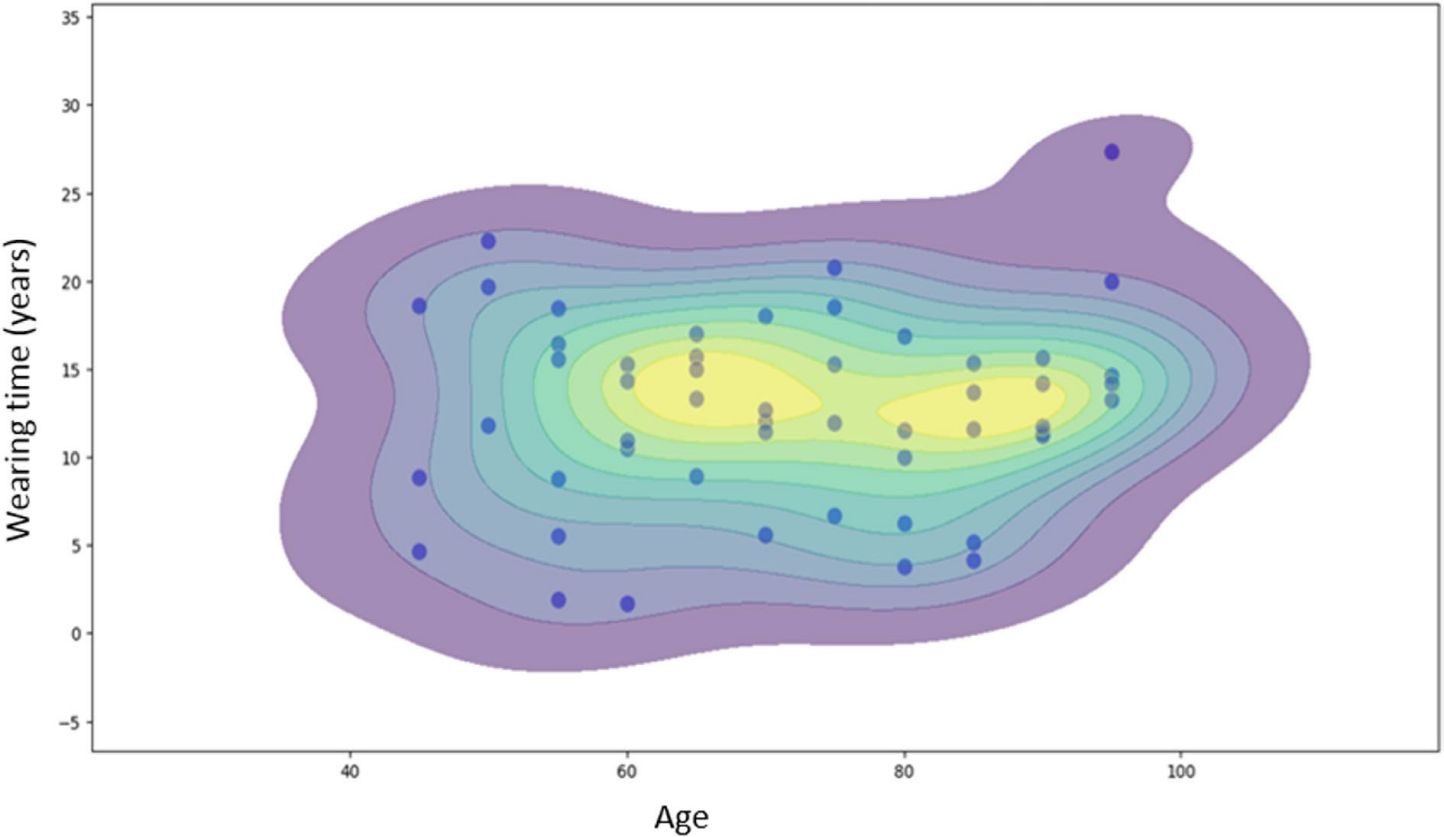
Scatter plots. Correlation between the age at implantation of the prosthesis and the patient's age at the onset of the disease. Colors represent the density of data points, with a gradient from purple (low density) to yellow (high density), indicating increasing concentration of overlapping observations.

From the data distribution, the pseudotumor in our population arose between the ages of 20 and 83 years, primarily developing in most cases after the age of 56 years. The average wearing time was 12 years (range: 11.17–14). We did not find a significant correlation between the two variables (*r* = 0.0022, *p* = 0.734) (Figure 1).

Out of 55 patients affected by pseudotumor, 42 (76%) had a primary implant, and 13 (24%) had a revision. The mean wearing time was 12.6 ± 0.73 years. The kind of bearing was: CoC in 10 patients (18%), MoM in 12 patients (22%), MoP in 30 patients (55%), and CoP in the last 3 patients (5%). The implant resulted loosened in 65% of cases (36/55): 75% (27/36) showed acetabular loosening, 8% (3/36) stem loosening, and 17% (6/36) had both stem and acetabular loosening. Implant related data are reported in Table 2.

**TABLE 2:** Implant related data.

Variable	Category	*N* (%)
Implant type	Primary	42 (76)
	Revision	13 (24)
Bearing	CoC	10 (18)
	MoM	12 (22)
	MoP	30 (55)
	CoP	3 (5)
Aseptic loosening	No	19 (35)
	Yes	36 (65)
Strategy of treatment	One stage	24 (44)
	Two stage	16 (29)
	Girdlestone	9 (16)
	Simil‐dair	4 (7)
	Hindquarter amputation	1 (2)
	Definitive spacer	1 (2)
Bacterial contamination	Negative	28 (51)
	Positive	18 (33)
	Not determined	9 (16)
Complication	Total Complications	10 (18)
	Surgical wound complication	1 (10)
	Abductor muscle dysfunctions	4 (40)
	Deep periprosthetic infection	1 (10)
	Hip dislocations	4 (30)
Mean Harris hip score 73.2 (95% CI 68.6–77.8)		

Abbreviations: CI, 95% interval of confidence; CoC, ceramic on ceramic; CoP, ceramic on polyethylene; MoM, metal on metal; MoP, metal on polyethylene.

Twenty‐four patients were treated in one‐stage strategy (44%), 16 in two‐stage strategy (29%), 9 in Girdlestone (16%), 4 in Simil‐dair (7%), 1 hindquarter amputation (2%), and 1 (2%) with definitive surgical spacer. Mean time to second stage treatment amounts to 6.4 months (95% CI 4.30–8.44). Considering the subset of one and two‐stage procedures (40/55), we reported 10 complications: 1 wound or deep infection, 4 hip prosthesis dislocations, 4 severe abductor dysfunction. The patients were clinically assessed in post‐revision settings using the Harris hip score (HHS) [14]. The post‐operative mean score was 73.2 (95% CI: 68.6–77.8).

Implant's follow‐up was available for 50 out of 55 patients: 4 patients were lost to follow‐up; one patient died immediately after surgery. Patients' mean survival time was 71.2 months (95% CI: 58.14–84.36). At the last follow‐up, 14 patients had died, with only two of them (14%) succumbing to complications related to sepsis following surgery. Overall patient's survival corresponded to 83% (95% CI: 0.68–0.92) and 67% (95% CI: 0.48–0.80), at 5 and 10 years.

### 3.2 One Stage and Two Stage Approaches at Comparison

**FIGURE 2:**
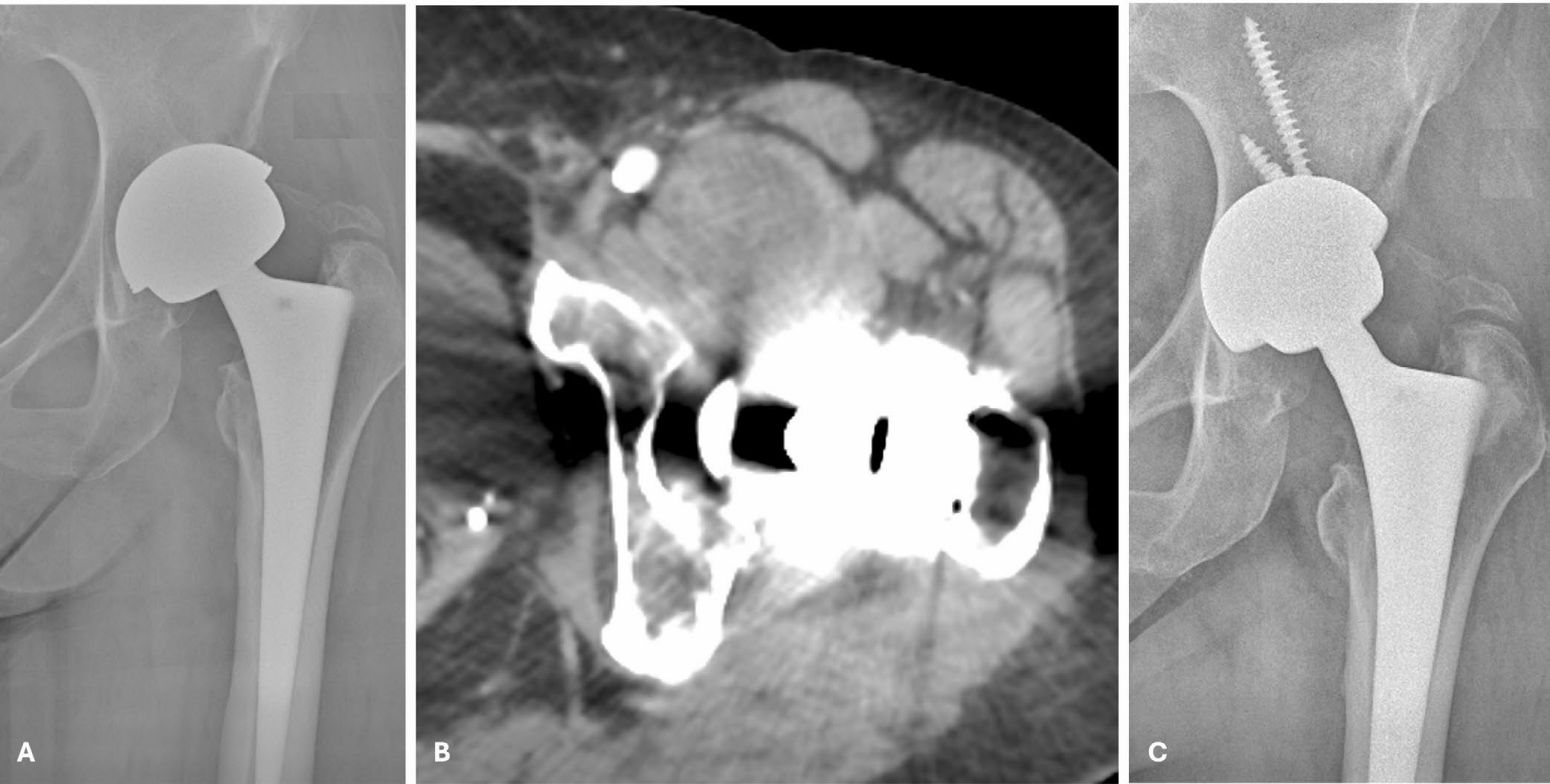
Imaging studies and surgical steps from a patient with a pseudotumor of the hip treated using a one‐stage approach. (A) Preoperative plain antero‐posterior X‐ray and (B) axial CT scan with contrast of a 58‐year‐old female patient treated 7 years earlier with a MoM hip prosthesis for primary hip arthrosis. Pre‐operative CRP was within normal range, and the stool testing was negative for bacteria. The patient was considered for a one‐stage approach. (C) Post‐operative plain antero‐posterior X‐ray taken 12 months after the acetabular revision.

**FIGURE 3:**
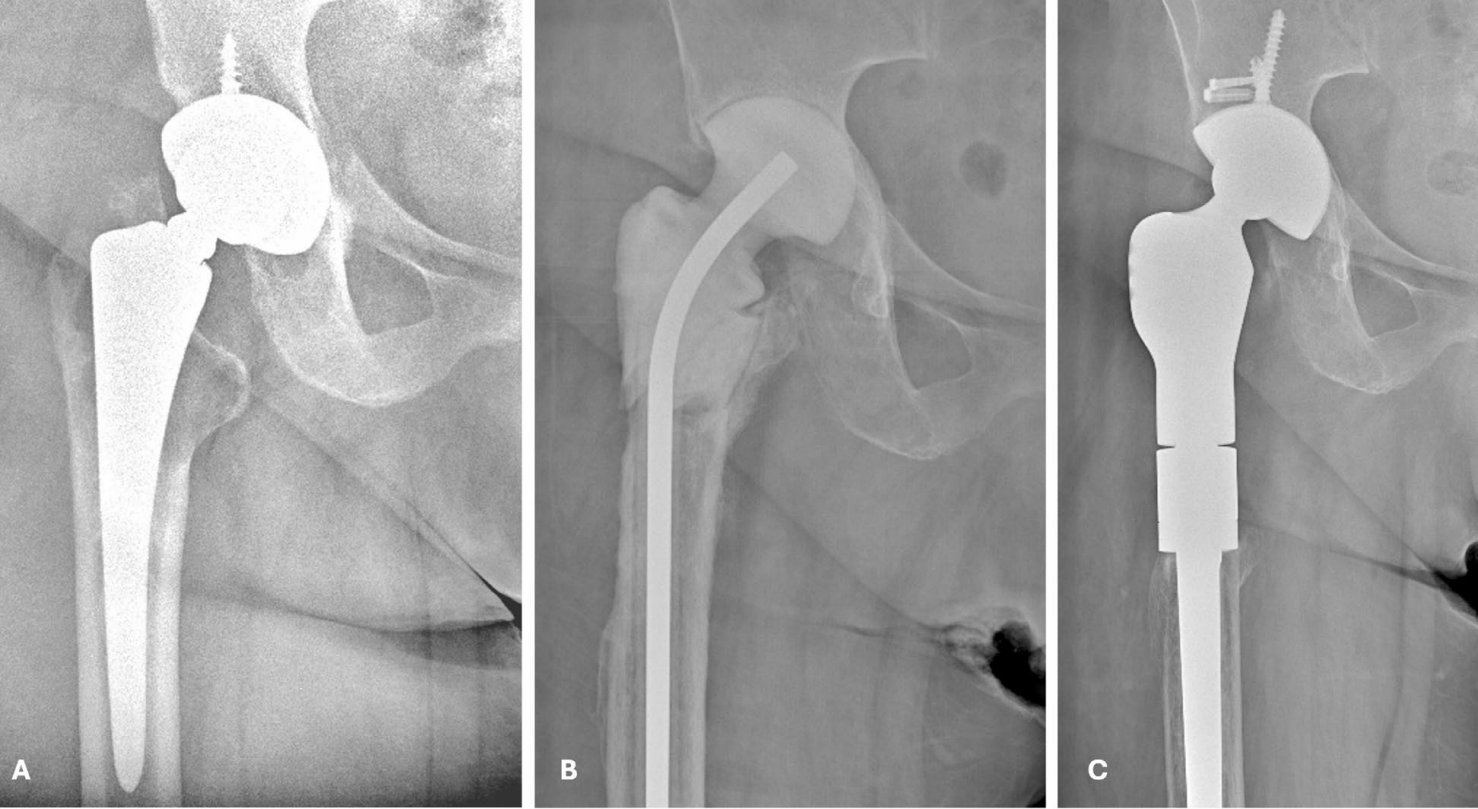
In (A), preoperative plain antero‐posterior X‐ray of a 77‐year‐old female patient treated 12 years earlier with a CoP hip prosthesis for primary hip arthrosis. A needle biopsy and stool culture were carried out to define the type of surgical approach. Due to the stool testing positive for bacteria, a two‐stage approach was preferred. (B) Post‐operative X‐ray. The primary hip prosthesis was removed, and a hip antibiotic‐loaded spacer was inserted. Intraoperative stool culture also tested positive for bacteria. The patient underwent antibiotic therapy. (C) Once the infection was controlled, a proximal femur mega‐prosthesis and a revision shell prosthesis were implanted.

A comparison of one‐stage and two‐stage revision strategies in 40 out of 55 cases revealed a statistically significant association with increased CRP values (*p* = 0.013), the presence of positive bacterial cultures (*p* = 0.010), and tumor size (*p* = 0.003). Specifically, 93% of patients with altered CRP values underwent a two‐stage procedure (Figure 2). Additionally, the two‐stage approach was predominantly used for tumor volume more than 500 cm^3^. Other factors, including post‐operative complications, did not show significant differences between the groups (Figure 3).

The overall complication rate was 20%, with specific rates of 12% for one‐stage and 31% for two‐stage surgical procedures (*p* = 0.15) (Table 3).

**TABLE 3:** Comparison between one‐stage and two‐stage revision.

Factor	Level	One‐stage	Two‐stage	*p*
Gender	Female	14 (58%)	11 (69%)	*Χ* ^2^ (1) = 0.44 *p* = 0.50
	Male	10 (42%)	5 (31%)	
Age	≤ 56	9 (38%)	5 (31%)	*Χ* ^2^ (1) = 0.16 *p* = 0.68
	> 56	15 (62%)	11 (69%)	
BMI	Normal	9 (38%)	5 (31%)	*Χ* ^2^ (1) = 1.29 *p* = 0.73
	Overweight	11 (46%)	6 (38%)	
	Obesity	1 (4%)	1 (6%)	
	Underweight	3 (12%)	4 (35%)	
CRP	Not altered	9 (45%)	1 (7%)	*Χ* ^2^ (1) = 6.17 *p* = 0.013
	Altered	11 (55%)	14 (93%)	
ASA	I	1 (4%)	1 (6%)	*Χ* ^2^ (1) = 1.66 *p* = 0.65
	II	15 (62%)	9 (56%)	
	III	8 (33%)	5 (31%)	
	IV	0	1 (6%)	
Size	< 573 cm^3^	20 (83%)	6 (38%)	*Χ* ^2^ (1) = 8.87 *p* = 0.003
	≥ 573 cm^3^	4 (17%)	10 (62%)	
Bacteria culture	Positive	3 (12%)	9 (56%)	*Χ* ^2^ (1) = 9.20 *p* = 0.010
	Negative	15 (62%)	6 (38%)	
	Not dated	6 (25%)	1 (6%)	
Complication	No	21 (88%)	11 (69%)	*Χ* ^2^ (1) = 2.109 *p* = 0.15
	Yes	3 (12%)	5 (31%)	

Abbreviations: ASA, American Society of Anaesthesiologist values; BMI, body max index; CI, 95% interval of confidence; CRP, C‐reactive protein.

In the subgroup of patients with elevated CRP, postoperative complications occurred in 6/30 (20%) patients, with 1/15 (6.7%) in the one‐stage revision group and 5/15 (33.3%) in the two‐stage revision group. Although there was a trend toward a higher complication rate in the two‐stage group, the difference was not statistically significant (Fisher's exact test *p* = 0.169).

### 3.3 Implant Survival Analysis

Considering the whole cohort of patients, the overall implant survival rate was 92% (95% CI: 0.68–0.97) and 80% (95% CI: 0.44–0.94) at 5 and 10 years respectively.

**FIGURE 4:**
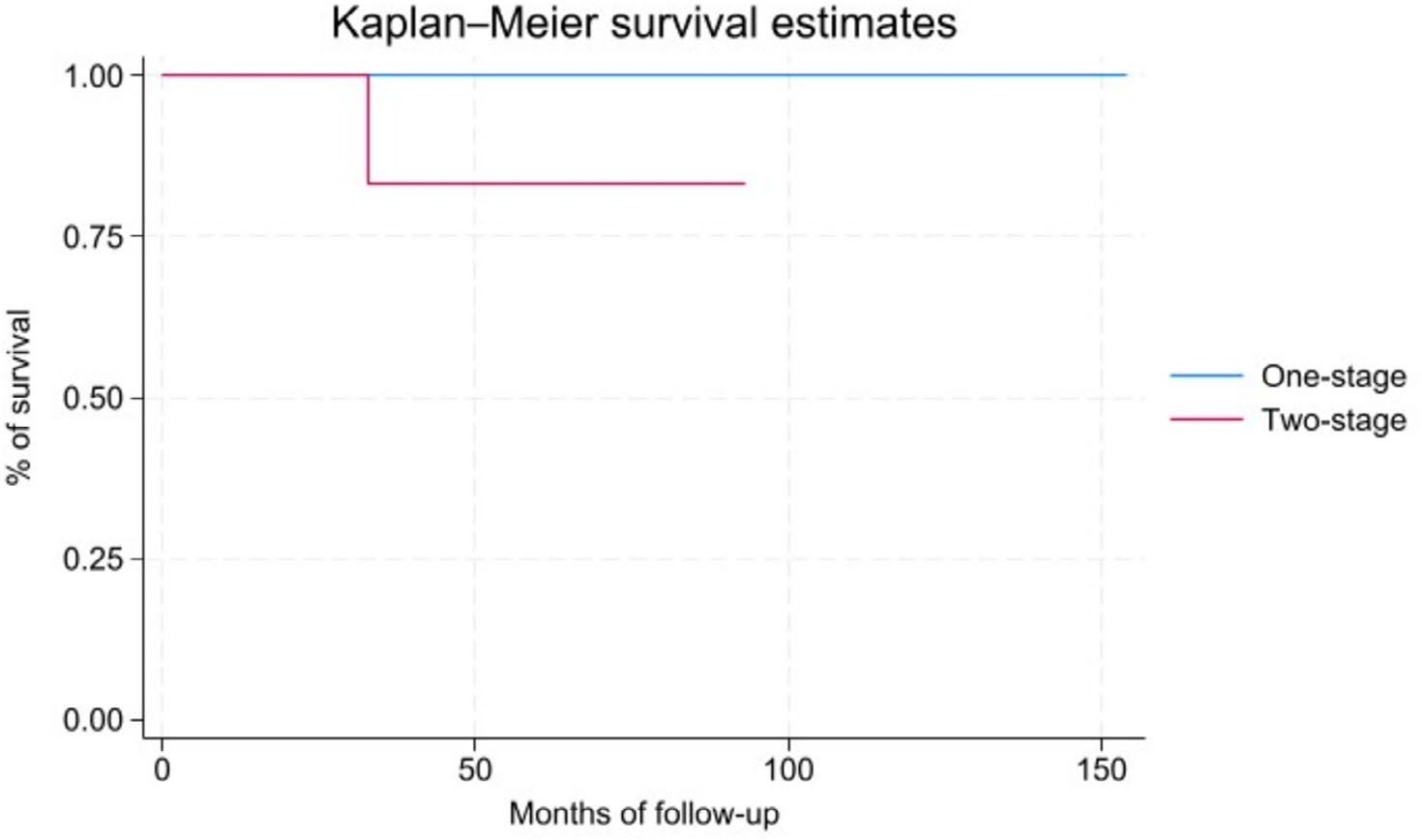
Kaplan–Meier survival curves illustrate the difference in survival between the one‐stage and two‐stage groups; however, this difference was not statistically significant.

The evaluation of implant survival focused exclusively on the cohort of patients who underwent either a one‐stage or two‐stage treatment strategy, comprising 40 out of 55 individuals. The average survival duration was recorded at 40 months (95% CI: 25–56). An overall implant survival rate of 93% (95% CI 0.61–0.99) was noted at both the 5‐ and 10‐year marks (Figure 4). No significant correlation was identified between clinical variables and implant survival in the univariate analysis (Table 4).

**TABLE 4:** Univariate analysis of variables associated with implant survival limited to patients treated with one‐stage or two‐stage strategy.

		*N* (%)	5‐year survival (%)	95% CI	Log‐rank test (*p*)
Gender	F	25 (62)	90	0.5–0.98	0.5456
	M	15 (38)	100	—	
Age	< 56 years	14 (35)	100	—	0.3496
	≥ 56 years	26 (65)	87	0.39–0.98	
CRP altered	No	10 (29)	100	—	0.5050
	Yes	25 (71)	88	0.43–0.98	
BMI category	Normo weight	13 (33)	87	0.39–0.98	0.9281
	Obesity I	3 (8)	—	—	
	Obesity II	4 (10)	100	—	
	Underweight	2 (5)	100	—	
	Overweight	18 (45)	100	—	
Complication	No	32 (80)	100	—	0.0973
	Yes	8 (20)	75	0.13–0.96	
Implant	Primary	32 (80)	92	0.57–0.98	0.6949
	Secondary	8 (20)	100		
Bearing	CoC	6 (15)	100	—	0.9189
	MoM	10 (25)	100	—	
	MoP	21 (53)	90	0.47–0.98	
	CoP	3 (7)	—		
Size	< 573 cm^3^	33 (60)	100	—	0.2207
	≥ 573 cm^3^	22 (40)	83	0.27–0.97	

Abbreviations: ASA, American Society of Anaesthesiologist values; BMI, body max index; CI, 95% interval of confidence; CoC, ceramic on ceramic; CoP, ceramic on polyethylene; CRP, C‐reactive protein; MoM, metal on metal; MoP, metal on polyethylene.

## 4 Discussion

A pseudotumor of the hip is described as a non‐malignant soft‐tissue mass arising around the hip joint following total hip arthroplasty. This condition often results from the accumulation of wear debris, especially in MoM implants, which can trigger an inflammatory response that leads to the formation of a pseudotumor [2]. There is increased interest in the incidence of pseudotumor, its impact on the decision to revise, and the outcomes of those revisions [6, 15].

In this context, the optimal surgical management remains controversial, particularly regarding the choice between one‐stage and two‐stage revision strategies.

The standard of care for treating pseudotumor is far from established, remaining a contentious topic among surgeons. Thus, considering that one‐stage or two‐stage surgery is controversial in the treatment of pseudotumor, we investigated which pre‐operative factors could drive the surgeon's choice. We provided a consecutive case series of 40 revision hips with a one‐stage or two‐stage strategy of pseudotumor resection and revision.

### 4.1 Main Findings of This Study

The main findings of this study are as follows: (i) surgical strategy was mainly influenced by tumor size and CRP levels; (ii) two‐stage procedures were more frequently used in patients with extensive disease and altered inflammatory markers; (iii) overall complication rate was 20%, lower than previously reported; and (iv) implant survival reached 92% at 5 and 10 years, with acceptable patient survival rates.

### 4.2 Patient Characteristics and Risk Profile

Demographically, pseudotumors are prevalently found in female elderly patients [13, 16]. In our series, we confirmed this trend, finding a preponderance of female patients (62%), older than 56 years (65%). We didn't find a significant correlation between the wearing time and age. Our series was characterized as well by 62% of patients with high BMI (greater than 25) and by 42% of them with mild or severe systemic disease (ASA III and IV), underscoring the fragility of this category of patients.

### 4.3 Inflammation, Infection, and Implant Type Distribution

Altered CRP values are diagnostically associated with the presence of bacterial infection [17]. CRP is one of the most published serum biomarkers utilized in the diagnosis of periprosthetic joint infection [18]. Other studies have investigated the prognostic role of CRP values in the detection of pseudotumor, especially due to the production of metal ions realized by MoM bearing [19, 20]. In our cohort of patients, we confirmed that infection is common among patients affected by pseudotumor, finding a 25% positivity to CRP among patients. Despite older data, where pseudotumor was mostly found in MoM hip prosthesis, in our study there was a prevalence of MoP bearings (52%), followed by MoM (25%), CoC (15%), and CoP (8%). This shift likely reflects the broader adoption of MoP implants in recent years and findings consistent with more recent literature [11, 17, 18, 19, 21].

### 4.4 Pathophysiology and Surgical Rationale

Pseudotumor can generate complications of different nature, like tissue damage, implant loosening, and chronic joint instability. Conversely, inflammatory condition may favor pseudotumor. This reciprocal action is pivotal for the definition of the best surgical strategy. Surgery should prevent eventual forthcoming complications getting to better and long‐lasting results.

### 4.5 Surgical Philosophy and Soft‐Tissue Management

In our clinical experience, we do not perform routine “oncologic” *en‐bloc* excision, but rather selective removal of cystic and necrotic components and pathological pseudocapsule when safely feasible. Preservation of the abductor mechanism and neurovascular structures was considered a key priority, as excessive debridement was associated with higher instability risk.

### 4.6 Complications and Surgical Outcomes

The excision of a pseudotumor, accompanied by either a total or partial prosthetic revision, is closely associated with a high incidence of severe postoperative complications, occurring in 50% of patients. This often necessitates further revision surgeries in 30% of these cases [22]. In our study we registered an overall complication rate of 20%, amounting to 12% and 31% for the on‐stage and two‐stage surgery procedures, respectively. This value is inferior to other rates already published [13, 22]. We tried to find an explanation for this result in the kind of surgery adopted, but we found no differences concerning complications between the two interventional procedures (*p* = 0.15). On the other hand, the revision strategy resulted to be associated with altered CRP values (*p* = 0.013), and with tumor size (*p* = 0.003); in particular, patients with extensive tumor and altered CRP values were prevalently treated with a two‐stage approach. Bacterial contamination was explored as well on pathological specimens, sent out to examine for infectious or inflammatory causes related to the patient's presentation and implant failure during the revision arthroplasty. This characterization of intraoperative specimens is essential during a diagnostic workup and was pivotal in diagnosing a pseudotumor.

### 4.7 Extent of Resection and Literature Comparison

Consistent with current evidence, we don't perform an “oncologic” *en‐bloc* excision of the pseudotumor. Instead, we adopt selective removal of cystic/necrotic components and the pathologic pseudocapsule when safely feasible, avoiding sacrifice of the abductor mechanism or hazardous neurovascular dissection. The shared principle is that the clinical course is primarily determined by eliminating the source of debris (revision of the offending components), whereas the extent of soft‐tissue debridement should be individualized according to morphology (cystic vs. solid), available planes, and the risk to abductor and neurovascular structures [23, 24, 25]. Extensive debridement has been associated with higher postoperative instability—particularly when compromising the abductors—whereas earlier revision, before severe soft‐tissue damage occurs, reduces complications and re‐revision rates. Accordingly, capsulo‐tendinous structures contributing to stability should be preserved whenever possible [9, 24]. One‐stage “radical” excision with conversion to non‐MoM bearings has been reported with satisfactory short‐ to mid‐term outcomes but relevant complications and possible recurrences; when the debris source is eliminated, recurrences seldom mandate re‐revision [26]. Conversely, for intrapelvic or critically adherent lesions, complete excision is often impractical and targeted debulking combined with joint revision remains acceptable [25].

### 4.8 Indications for One‐Stage Versus Two‐Stage Revision

Consistent with earlier findings, we found that in the case of extended tumors and existing infection, a two‐stage approach is preferable, either to minimize the duration for each surgery or to optimize the individual design of revision hip implants [13, 25]. This evaluation is particularly reliable considering the most innovative reconstruction procedures based on custom 3D‐printing. In this scenario, a two‐stage approach can be planned with a more personalized hip implant [27, 28]. Finally, we recorded no differences in terms of functional outcomes between the two interventional procedures. However, this result is limited to the post‐operative setting, since we did not register the pre‐operative score, and we could not compare it with the post‐operative one.

The interval between the first and second‐stage surgeries was determined not only by inflammatory markers such as CRP but also by mechanical and functional considerations. In patients with significant acetabular bone loss, the use of temporary cement spacers posed challenges, including the risk of dislocation and limitations in weight‐bearing. As a result, early mobilization was often restricted, and rehabilitation protocols had to be carefully adapted to ensure joint protection while maintaining muscle function. These factors influenced the timing of the second‐stage revision, which was scheduled based on a combination of biochemical resolution, soft tissue recovery, and mechanical stability. This underscores the importance of a patient‐specific approach and close multidisciplinary monitoring during the inter‐stage period.

### 4.9 Outcomes

The treatment of this pathology usually gives poor results with a high intervention and complication rate [25, 29]. In the present study overall implant longevity reached a mean survival time of 35 months (95% CI: 23.4–46). At 5‐ and 10‐years implant survival rate was 92% (95% CI: 0.68–0.97) and 80% (95% CI: 0.44–0.94), respectively. The survival rate of implants within the cohort of patients who underwent either a one‐stage or two‐stage surgical approach is 93% (95% CI: 0.61–0.99) at both 5 and 10 years following the implantation. Moreover, although multivariable logistic regression could have provided further insights into predictive factors, the small sample size and low event rate precluded its use without risking model overfitting. We therefore limited our analysis to descriptive and univariate methods and recommend that future studies with larger cohorts incorporate multivariable models to better identify independent predictors. Finally, overall patient's survival corresponded to 83% (95% CI: 0.68–0.92) and 67% (95% CI: 0.48–0.80) at 5 and 10 years, confirming that treatment of pseudotumor leads to a fair prognosis. However, the cause of death for comorbidities in 12 out of 14 cases underlines the intrinsic fragility of this category of patients.

### 4.10 Limitation and Strengths

The present study has several limitations. First, this cohort represents a heterogeneous population with multiple comorbidities and potential confounders that may affect postoperative outcomes. Selection bias is also likely due to the patient selection criteria used at our institution.

A further limitation is the retrospective design, which restricted data availability; specifically, only C‐reactive protein (CRP) levels were consistently available, whereas other inflammatory markers such as erythrocyte sedimentation rate (ESR) and procalcitonin (PCT), which could enhance infection assessment, were not routinely measured.

In addition, the small sample size limited the power to detect associations and precluded reliable multivariable analysis due to the risk of overfitting. Similarly, low case numbers and uneven group distribution prevented meaningful comparisons between specific subgroups.

Survival analyses are also limited by the decreasing number of patients at risk at longer follow‐up intervals, particularly beyond 5–10 years, which may affect the precision of long‐term survival estimates. Moreover, the wide study period (2004–2023) resulted in heterogeneous follow‐up durations, with shorter observation times for more recently treated patients.

Lastly, it cannot be excluded that deceased patients experienced local disease progression after the treatment.

Despite these limitations, this represents a consecutive real‐world series with clinically relevant mid‐ to long‐term follow‐up, providing useful insights into a rare condition.

## 5 Conclusions

**FIGURE 5:**
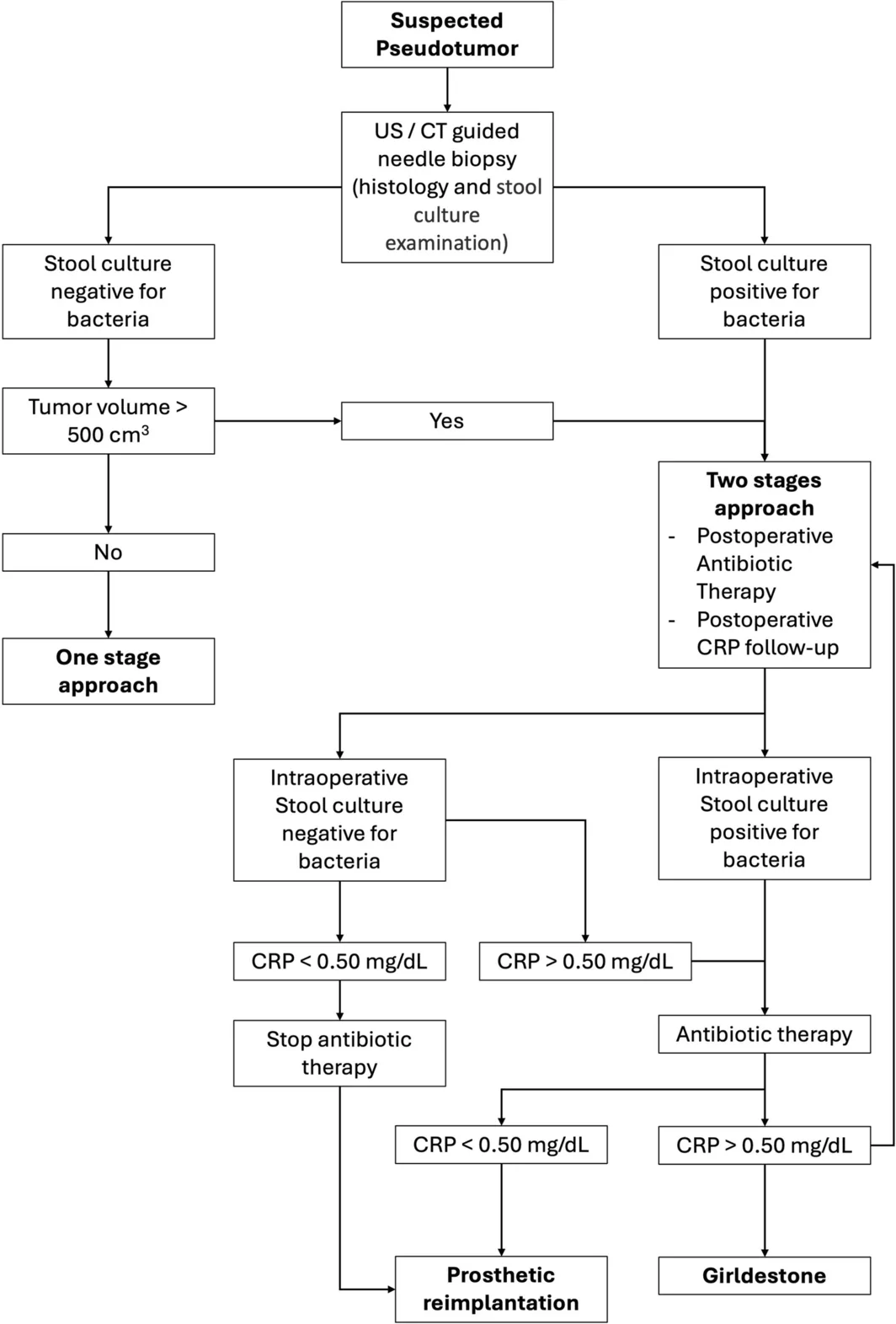
Literature‐based flow‐chart for treating hip pseudotumor.

The surgical revision treatment of patients affected by pseudotumor guarantees a good functional result with low risk of failure of the new implant. However, pseudotumor requires a systematic and efficient surgical approach to be quickly and successfully resolved. According to the data reported in the literature, we propose literature‐based guidelines for treating hip pseudotumor (Figure 5). As such, a comprehensive laboratory evaluation including CRP was all required before reaching a definitive diagnosis. We recommend a two‐stage approach in the presence of bigger lesions and ongoing infection to reduce the surgical time for each surgery and allow a more fitting design of a revision implant. Moreover, the increasing use of custom 3D‐printed implants supports the choice of a two‐stage procedure.

## Author Contributions

**Anna Mammone:** data curation. **Roberta Laranga:** conceptualization, formal analysis, writing – original draft, writing – review and editing. **Davide Maria Donati:** supervision. **Giuseppe Bianchi:** data curation, supervision, validation, methodology, conceptualization, project administration. **Eric Lodewijk Staals:** data curation. **Laura Campanacci:** data curation. **Luca Cevolani:** writing – original draft, writing – review and editing, conceptualization, investigation, methodology, data curation, supervision, project administration. **Barbara Dozza:** data curation.

## Funding

The authors have nothing to report.

## Ethics Statement

The Ethics Committee of the Hospital approved the study. A statement of the location where the work was performed (only if authors are from multiple institutions). The study was performed at the Rizzoli Orthopedic Institute, via Pupilli 1, 40,136 Bologna (Italy).

## Consent

The patient was informed that data concerning the case would be submitted for publication, and a Statement of Informed Consent was achieved.

## Conflicts of Interest

The authors declare no conflicts of interest.

## Data Availability

The data that support the findings of this study are available on request from the corresponding author. The data are not publicly available due to privacy or ethical restrictions.
